# Encapsulation of Living Leishmania Promastigotes in Artificial Lipid Vacuoles

**DOI:** 10.1371/journal.pone.0134925

**Published:** 2015-08-04

**Authors:** Carlos E. S. Guedes, Jose G. B. Lima, Emmanuèle Helfer, Patricia S. T. Veras, Annie Viallat

**Affiliations:** 1 Centro de Pesquisas Gonçalo Moniz, Fiocruz, Laboratório de Patologia e Biointervenção, Rua Waldemar Falcão, 121, Candeal, Salvador, Bahia, Brazil; 2 Aix-Marseille Université, CNRS, CINaM UMR 7325, 13288, Marseille, France; INRS - Institut Armand Frappier, CANADA

## Abstract

After phagocytosis by mammalian macrophages, promastigote forms of *Leishmania* parasites settle inside intracellular parasitophorous vacuoles (PVs) in which they transform into amastigote forms and replicate. Here, using a variant of the ‘inverted emulsion’ method, we succeeded in encapsulating living *L*. *amazonensis* parasites in giant artificial liposomes that serve as model PVs. We were able to control the size of liposomes, the pH and the composition of their internal volume, and the number of internalized parasites per liposome. *L*. *amazonensis* promastigotes encapsulated in liposomes filled with RPMI-Dextran solution at pH 7.5 or 6.5 survived up to 96 h at 24°C. At 37°C and pH 5.5, parasites survived 48h. This method paves the way to identifying certain effectors secreted by the parasite and to unraveling specific mechanisms of fusion between the PV and intracellular vesicles of the host cell. This method will also facilitate the study of the temporal evolution of biophysical properties of the PV during its maturation.

## Introduction

Obligate intracellular parasites such as *Leishmania* spp., *Trypanosoma cruzi* and *Toxoplasma gondii*, agents of major diseases, are internalized by host cells through an endocytic process [1–4]. During the internalization process, parasites are surrounded by a membrane that originates from the plasma membrane of the host cell. This membrane delimits a vacuole in the host cell, named parasitophorous vacuole (PV), inside which the parasites settle individually or in groups. The PV protects the internalized parasites from the host cell environment. PVs are gradually adapted by the action of specific molecules secreted by the parasites (such as glycoconjugates, lysosomal proteases and ions) in order to create a suitable environment for their survival and their multiplication [5–7]. For instance, *Leishmania* parasites manage to regulate fusion events between their PVs and organelles of the host macrophage. This allows modification of the composition of PV membranes, increase of their size and supply of nutrients to the internalized parasites [8,9]. To identify and understand the role that molecular and biophysical parameters play in PV adaptation, the PV needs to be in controlled physical and biochemical environments. It is possible, for example, to reconstitute the interactions of PVs with small controlled synthetic vesicles or with vesicles extracted from the host cells. However, the isolation of PVs containing living *Leishmania* parasites from the host cell meets technical difficulties [10–12] that impede their easy manipulation *ex-vivo*.

In this study, we report the first attempt to encapsulate living *Leishmania* into giant liposomes, which are model systems that have previously successfully served as minimal systems to study biological functions [13]. We show that living *Leishmania* parasites (specie *L*. *amazonensis*) can be encapsulated in artificial giant unilamellar liposomes of controlled size and composition, and remain alive for several days. These artificial PVs containing parasites are suspended in a glucose solution where they can be easily individually handled and submitted to various physical or biochemical environments. They can be put in contact with specific molecules or vesicles and submitted to external forces, various temperatures and pH. The method of liposome preparation is simple and does not require specific costly equipment. It can be easily used in parasitology laboratories. However, it is not a mass production method. Only a few hundreds of liposomes encapsulating parasites are produced during an experiment. The method is thus well adapted to the handling of individual artificial PVs and their observation using optical microscopy.

A standard method used to encapsulate materials within giant liposomes is the electroformation technique [14, 15]. However, it does not enable encapsulation of parasites, which continuously move in the suspension and remain outside the liposomes, which swell from the surface of the electroformation device. Other methods, such as double emulsion and continuous droplet interface crossing encapsulation (cDICE) [16,17] have proved to be efficient for encapsulating a variety of particles or cells. However, these methods use centrifugation steps that require an accurate matching of density of parasites and of the medium in which they are suspended. As we found a large density variability among individual *L*. *amazonensis*, this matching was not possible and the yield of these methods was extremely low.

Here, we prepared artificial PVs encapsulating living *L*. *amazonensis* parasites in their promastigote form by adapting a new method introduced by Yanagisawa et al. [18] based on the inverted emulsion technique. We first prepared a micro-emulsion made of micro droplets containing parasites in their culture medium in suspension in a continuous lipid-oil solution. The lipids stabilized the droplets by forming a monolayer at their surface, which formed the inner leaflet of the future liposome. Then the micro-emulsion was deposited on the top of an oil phase placed above an aqueous phase. The droplets gently sedimented in oil and then across the lipid-oil-water interface where they became coated by a second lipid monolayer and therefore turned into liposomes. The liposomes further sedimented through the aqueous phase. Each liposome encapsulated one or several parasites suspended in culture medium.

## Materials and Methods

### Materials

Lipids 1,2-dioleoyl-sn-glycero-3-phosphoethanolamine-N-[methoxy(polyethylene glycol)-2000] (PE-PEG); 1,2-dilauroyl-sn-glycero-3-ethylphosphocholine (EPC); 1,2-dioleoyl-sn-glycero-3-phosphocholine (DOPC); 1,2-dioleoyl-sn-glycerol-3-phosphoethanolamine (DOPE) and 1,2-dioleoyl-sn-glycerol-3-phosphor-L-serine (DOPS) were purchased from AVANTI polar lipids Inc. Glucose, Dextran 6,000, citric acid, Schneider’s medium, and mineral oil were acquired from Sigma-Aldrich. RPMI culture medium, foetal bovine serum (FBS), penicillin-streptomycin (P/S) antibiotics, and calcein AM were purchased from Life Technologies.

### Lipid-oil solution

The lipid-oil solution was prepared under N_2_ atmosphere, with humidity lower than 10% using a portable inflatable glove bag. We used two types of lipid mixtures to prepare the lipid-oil solution: a neutral EPC and PE-PEG mixture (98:2) and a charged DOPC, DOPE, DOPS mixture (50:25:25). The mixtures were dissolved in chloroform:methanol (9:1) at a total concentration of 100 mM. Then, 80 μl of the lipid solution was further diluted in 300 μl of chloroform:methanol in a 25 ml flask. The solvent was evaporated under primary vacuum for 1 h to obtain a lipid film at the bottom of the flask. Then, 20 ml of mineral oil were added and subjected to vacuum for additional one hour. Finally, the lipid-oil solution was sonicated for 20 min in an ultrasonicator at 40°C to obtain a stable lipid in oil solution at 0.4 mM. The solution was kept at room temperature and was used to prepare liposomes up to two weeks after preparation.

### *L*. *amazonensis* parasites

*L*. *amazonensis* parasites were cultivated in Schneider’s medium supplemented with 10% FBS and 1% P/S at 24°C. When the culture reached the stationary phase, it was washed three times in RPMI medium and re-suspended in different media for encapsulation. *L*. *amazonensis* parasites were in their flagellar motile promastigote form.

### Parasite suspension

We prepared three solutions in which parasites were suspended and encapsulated within giant liposomes: sucrose solution (360 mOsm), RPMI and RPMI-dextran solution. The latter was prepared to match the solution density to the parasite mean density. RPMI-Dextran was prepared by diluting RPMI with 20% volume of deionized water and adding Dextran 6,000 to reach a Dextran concentration of 20% (w/v). Finally, 10% FBS and 1% P/S were added to the solution and the pH was adjusted with citric acid. Lipid membranes are impermeable to ions so the pH was maintained inside liposomes. The final solution osmolality was adjusted to 360–365 mOsm by using an osmometer (Löser). Finally, 10^6^ to 10^7^ parasites were suspended in 50 μl of each of the prepared solutions.

### Production of micro-emulsion precursor medium

The micro-emulsion precursor medium was made by emulsifying 50 μl of sucrose, RPMI or RPMI-Dextran containing *L*. *amazonensis* promastigotes into the lipid-oil solution. The emulsion was produced by 30 successive aspirations and releases of the solution using a 1 ml-micropipette.

### Preparation of parasite containing liposomes

**Fig 1:**
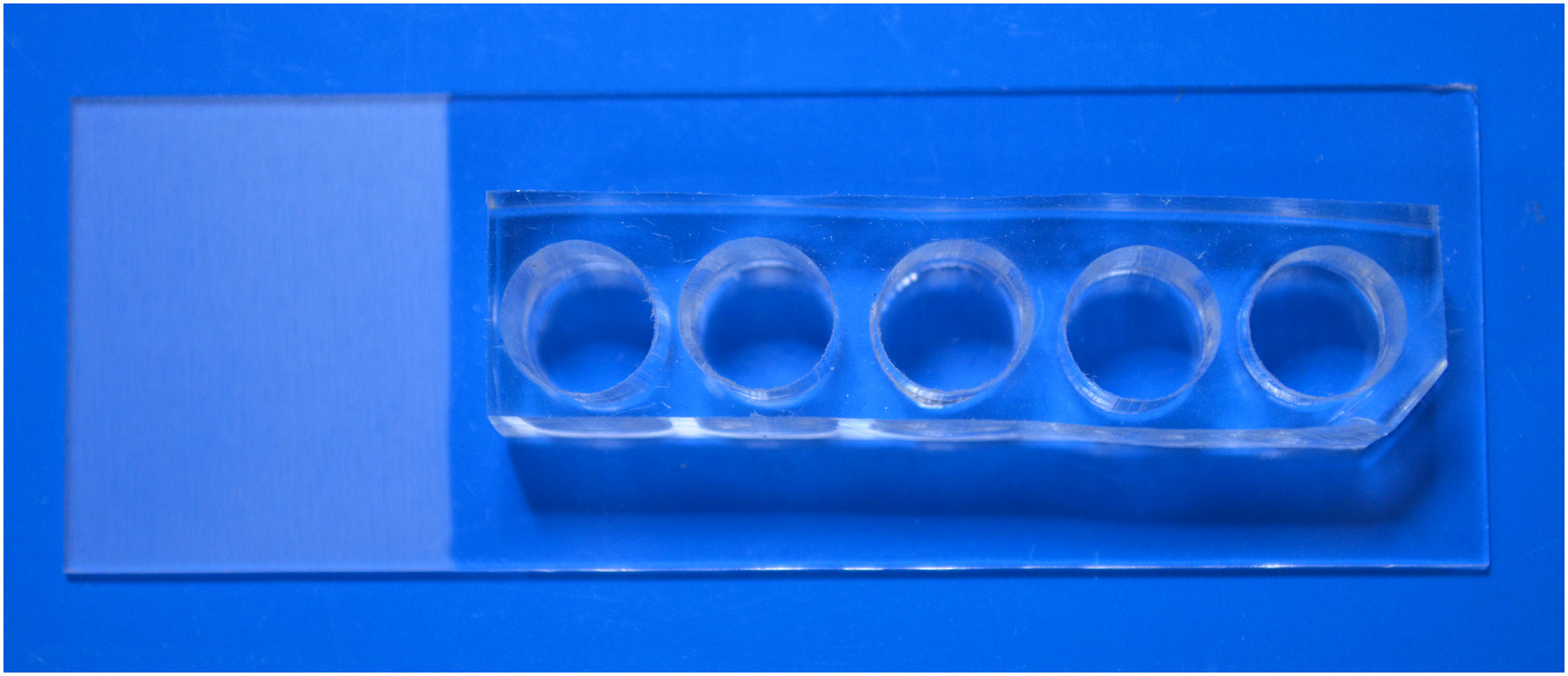
Picture of the homemade PDMS chambers for liposome production. The chamber size is 8 mm in diameter and 10mm in height). PDMS firmly adheres on a glass slide.

Liposomes containing living *L*. *amazonensis* promastigotes were prepared in homemade PDMS (polydimethylsiloxane) chambers consisting in cylindrical holes (8 mm in diameter) dug in a PDMS sheet (10 mm thick) fixed on a glass slide (see Fig 1). Immediately before use, the PDMS chambers were sterilized by direct UV exposition for 20 min. Each chamber was then filled with 100 μl of glucose solution (365 mOsm) supplemented with 1% P/S and covered with 100 μl of the lipid-oil solution. After one hour, it was considered that a homogenous lipid monolayer had time to form at the lipid-oil/glucose solution interface. Then, 50 μl of the precursor micro-emulsion containing parasites were added in the oil phase at the top of the chamber. While sedimenting throughout the chamber the micro droplets containing parasites crossed the lipid-oil/glucose solution interface and turned into liposomes before gently settling in the glucose solution at the bottom of the chambers. Liposomes were then collected with a micropipette, transferred to culture plates (Multiwell, Becton Dickinson) containing 2 ml of glucose solution and stored in an incubator at the chosen temperature under controlled CO_2_ atmosphere.

### Quantification of parasite viability

Parasite viability was studied at 24°C, which is the suitable temperature to maintain promastigotes in axenic culture, and at 37°C, temperature at which promastigotes transform into amastigotes. Twice a day, we observed encapsulated parasites to check their flagellum mobility. We also used Calcein AM to label living parasites. 1 μM of calcein was added to 1 mL of the *L*. *amazonensis*-containing liposomes suspension. After 2 hours, the total number of parasites (from 50 to 90 parasites) and the number of fluorescent parasites were counted in at least 40 liposomes randomly selected.

### Microscopy observations

Liposomes were observed with an inverted microscope (OLYMPUS IMT-2) in bright field or phase contrast under magnification of 200, 400 and 600 times. Real time movies were recorded with a CCD camera (COHU). Fluorescence microscopy was performed with an inverted fluorescence microscope (Nikon Eclipse Ti) and pictures were taken using a cooled fluorescence camera (Andor).

## Results

### Liposomes encapsulating *L*. *amazonensis*

The process of liposome formation at the lipid-oil/glucose solution interface was observed by optical microscopy. After sedimentation in the lipid-oil solution, parasite-containing micro droplets arrived at the interface between the lipid-oil and the glucose solutions. We observed that some micro droplets remained at the interface where they broke and released their parasites, but many droplets spontaneously crossed the lipid monolayer, driven by the small difference in molar density. A light tap on the table on which the PDMS chambers were placed helped the emulsion droplets to pass through the interface. During this passage, the lipid monolayer at the interface between the lipid-oil solution and the glucose solution wrapped each crossing micro droplet, which was therefore converted into a liposome. After 20 minutes, hundreds of liposomes had settled at the bottom of the chamber (S1 Movie). The composition of the two layers of the lipid bilayer of the liposomes is believed to be identical since the same lipid-oil solution was used to prepare the emulsion (which gives the internal lipid layer of the liposomes) and to form the interface between the lipid-oil solution and the glucose solution in the PDMS chambers (which gives the external lipid layer of the liposomes).

**Fig 2:**
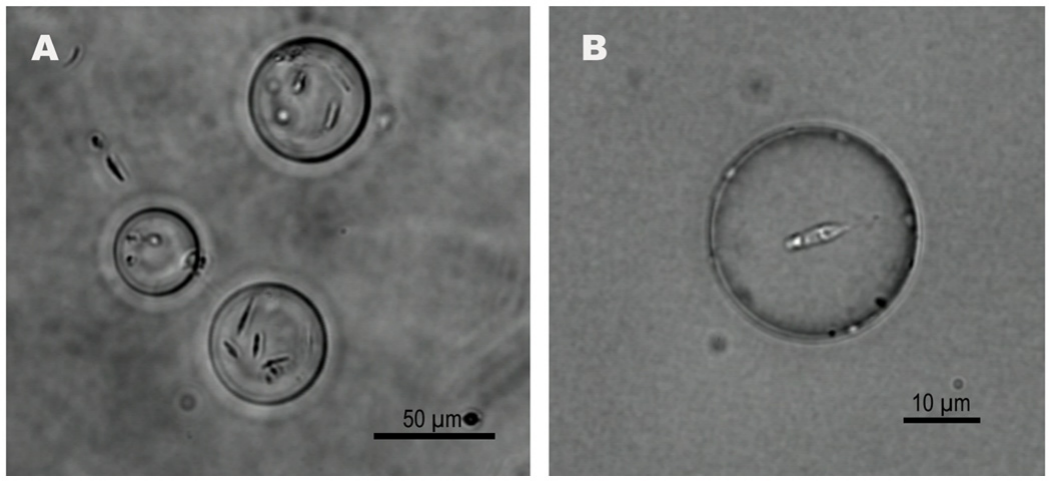
Liposomes encapsulating *L*. *amazonensis* promastigotes. The liposome membrane is composed of a EPC-PEG lipid mixture. Five million of *L*. *amazonensis* promastigotes per ml were used for the encapsulation process. (A) Liposomes containing multiple parasites. (B) Liposome with a single parasite with its flagellum at the right part of the parasite. Bright field images.

We successfully encapsulated *L*. *amazonensis* promastigotes inside EPC-PE-PEG liposomes containing sucrose, RPMI or RPMI-Dextran solutions. The RPMI-Dextran solution yields the highest encapsulation efficiency because its density matched that of parasites (see Fig 2). We also encapsulated *L*. *amazonensis* promastigotes in charged DOPC-DOPE-DOPS liposomes containing RPMI-Dextran solution. In this case, the divalent ions (such as Ca^2+^) released in the glucose solution after breakage of some liposomes at the interface interacted with the negatively charged lipids of the interface and of the liposome membrane. They mediated an attractive lipid-lipid interaction resulting in aggregation and sequestration of the liposomes at the lipid-oil/glucose solution interface. Addition of 15mM EDTA to the glucose solution enabled the detachment of liposomes from the interface and subsequent sedimentation toward the bottom of the chamber.

**Fig 3:**
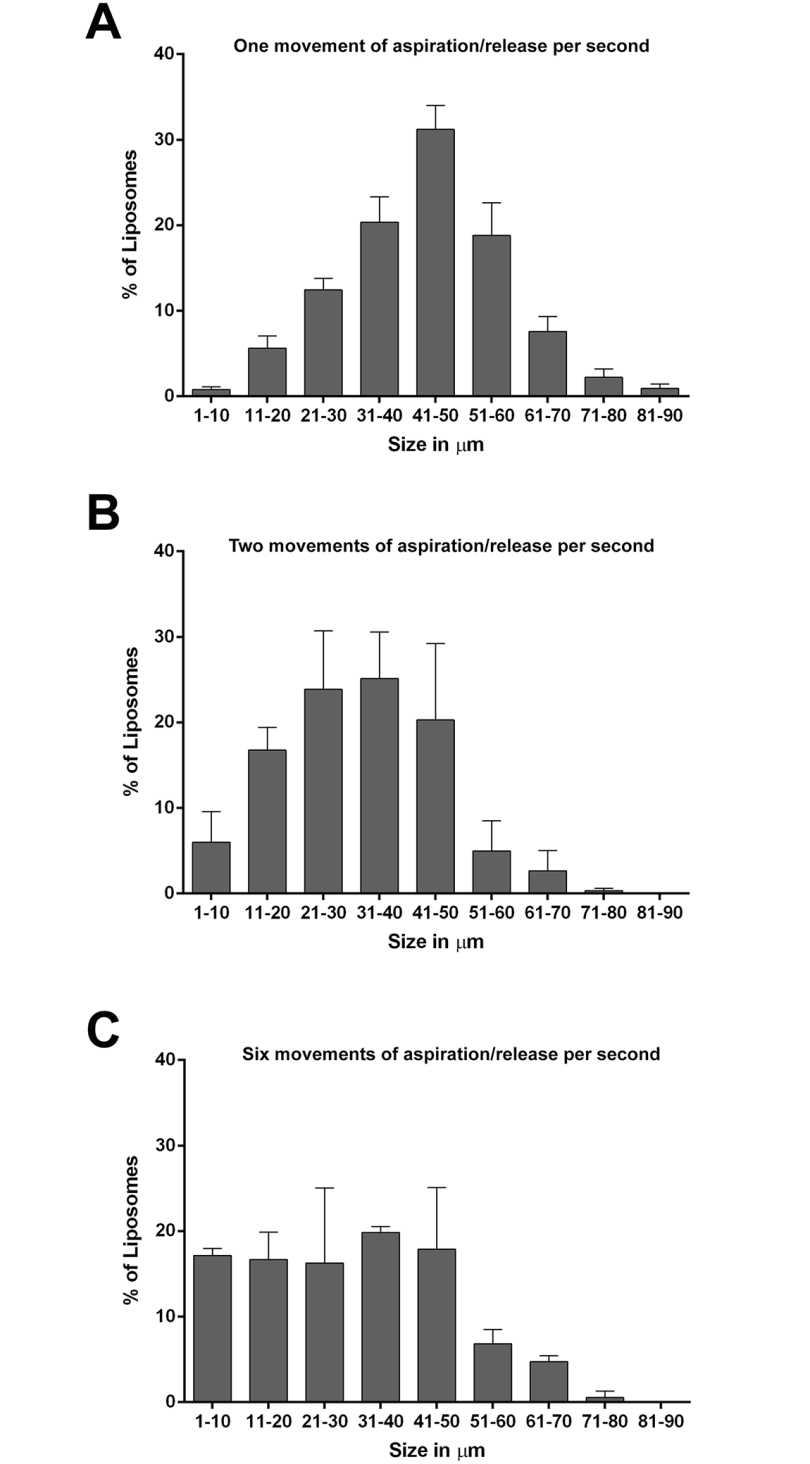
Size distribution of liposomes as function of aspiration/release speed. The micrp-emulsions were prepared by 30 aspiration/release cycles of the parasite suspension in a lipid-oil phase using a 1-ml micropipette. (A) one aspiration/release per second; (B) two aspiration/release per second; (C) six aspiration/release per second.

The size distribution of liposomes depended on the pipetting speed used to produce micro-emulsion as shown in Fig 3. The average liposome size decreased with the mixing speed. Fast aspiration/release (6 cycles / sec) produced droplets mainly ranging from 10 to 50 μm, each of them containing a few number of parasites. Slow aspiration/release (1 cycle / sec) produced larger droplets (20–70 μm) with dozens of *L*. *amazonensis* parasites inside them. In all cases, the liposome sizes were in a relevant range to mimic PVs. In the following, we focused on liposomes obtained with a micro-emulsion prepared by 30 successive aspirations and releases of the parasite suspension with a pipetting speed of two aspiration/release per second. These conditions led to liposomes ranging mainly from 21 to 50 μm which satisfactorily mimic PV sizes.

The number of encapsulated parasites per liposome increased with the parasite concentration in RPMI and RPMI-dextran solutions. Typically, we prepared liposomes containing from one to a dozen living promastigotes.

Parasites suspended in RPMI-Dextran solution were distributed within the whole liposome volume and freely swim within the liposome volume. In some cases, we observed an interaction between the flagellum of the parasite and the liposome membrane. We thus observed movements of the membrane induced by the movement of the flagellum. In rare cases, the tip of the parasite flagellum was stuck on the membrane, which, however, did not break.

### Parasite survival

In-vivo, *L*. *amazonensis* promastigote forms transform into the non-motile aflagellated amastigote form inside the PV, where they are able to survive and replicate within the macrophages. The time course of promastigote-to-amastigote differentiation occurs at 37°C and at acidic pH through four phases: signal perception (0–5 h after exposure); movement cessation and aggregation (5–10 h); amastigote morphogenesis (10–24 h); and maturation (24–120 h) [19]. In the present study, we focused on evaluating the viability of parasites within artificial PVs in the initial stages of intracellular parasite forms. It was beyond the scope of this paper to obtain a full transformation of parasites into the amastigote form. Indeed, the *in-vivo* conditions for amastigote transformation are not fully known, for example in terms of required molecules and of dynamics of their supply, of temporal evolution of pH and of PV’s size, etc.

The viability of parasites was ascertained by two ways. First, by observing their mobility and the motion of their flagellum. This method probes only the viability of the motile promastigote form. Second, by using Calcein AM which specifically labels living cells, thus both promastigote and amastigotes.

We first observed that isolated parasites incubated in RPMI-Dextran solution in culture chambers remained mobile and therefore alive during at least four days at pH 7.5 at 24°C. Because of the high viscosity of RPMI-Dextran solution, parasites moved more slowly in RPMI-Dextran suspensions than in pure RPMI medium.

As the pH of the PV is known to progressively acidify *in vivo* and decrease to 5.5 within 30 minutes after parasite uptake, we probed the lifetime of parasites in liposomes in RPMI-Dextran solutions first at pH 5.5, 6.5 and 7.5 at 24°C, and also under physiological conditions at pH 5.5 at 37°C. In this latter case, promastigotes are susceptible to start their transformation into amastigotes.

We observed promastigote motion inside artificial liposomes at 24°C. Most *L*. *amazonensis* promastigotes survived 48 h at pH 5.5 (S2 Movie), and 96 h at pH 6.5 and 7.5 (S3 Movie). When a liposome broke, the encapsulated parasites were released in the glucose solution and were not able to survive more than a few hours. Encapsulation in the liposomes therefore protected the parasites from the outer solution and enabled them to survive for several days.

**Fig 4:**
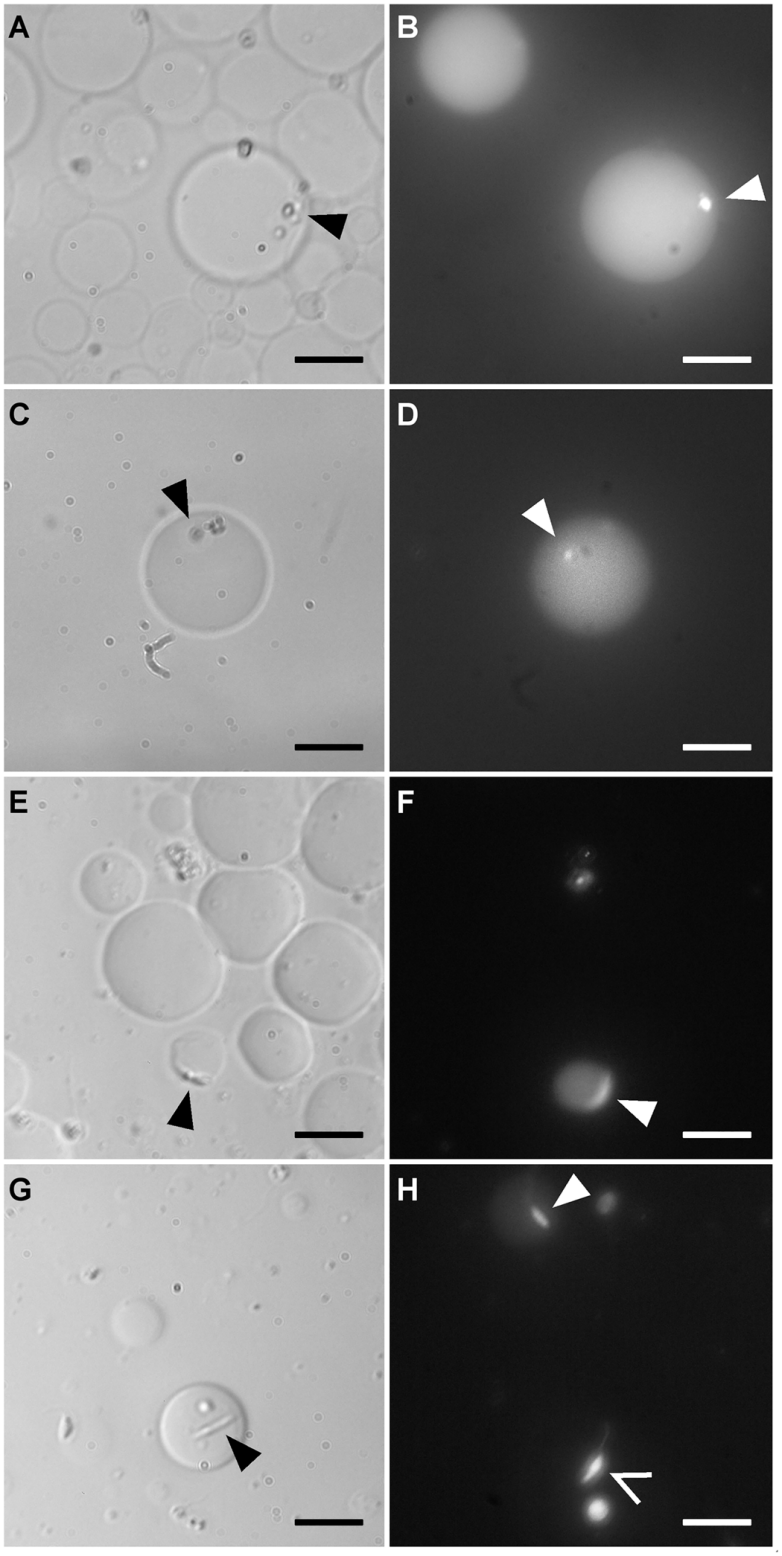
Observation of living *L*. *amazonensis* in liposomes. Liposomes containing parasites in RPMI-Dextran were incubated with Calcein before observation in phase contrast (left images) and fluorescence microscopy (right images). Liposomes are fluorescent due to the encapsulated living parasites (arrowhead) which expelled fluorescent Calcein after their uptake. (A-B and C-D) after 48h at pH 5.5 at 37°C; A and B: same field; C and D: same field. (E-F, G and H) after 48h at pH 7.5 at 24°C; E and F: same field. Promastigotes present elongated shapes. The empty arrow in H shows a living parasite, whose liposome had just broken. The flagellum is visible. Scale bar: 10 μm.

In order to quantitatively assess the survival rate of parasites within liposomes, we used the membrane-permeant live-cell labeling dye, Calcein AM. Upon entering a living organism, the non-fluorescent dye is converted to green-fluorescent and remains trapped in the organism. When added in the suspension of parasite-containing liposomes, Calcein diffused through the liposome membrane, entered in internalized parasites and became fluorescent in living parasites (Fig 4). Interestingly, we observed that *L*. *amazonensis* was able to expel fluorescent calcein in the host liposome, which also became fluorescent as shown in Fig 4B.

**Fig 5:**
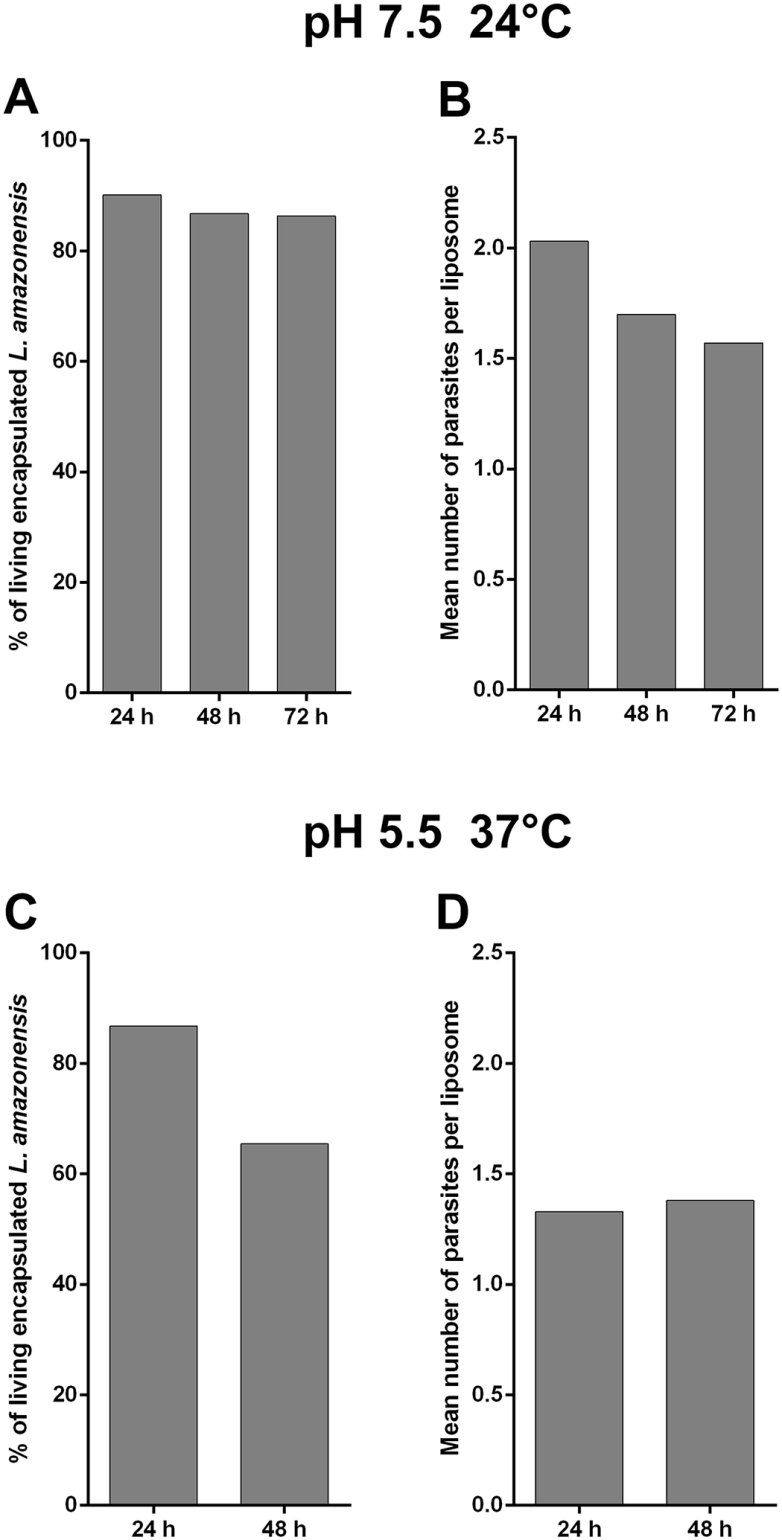
*L*. *amazonensis* viability in liposomes. Parasites in RPMI-dextran were encapsulated in liposomes and the living parasites were counted as function of time. (A) Average percentage of living encapsulated parasites. (B) Average number of living parasites per liposome after 24, 48 and 72h at pH 7.5 at 24°C. (C,D) Same parameters as in A and B after 24 and 48h at pH 5.5 at 37°C.

At 24°C and pH 7.5, the survival percentage of parasites trapped inside liposomes containing RPMI-dextran was 90.2, 87.7 and 86.3% after 24 h, 48 h and 72 h, respectively (Fig 5A). The average number of living parasites per liposome slightly decreases with time, from 2 to 1.7 and 1.5 after 24, 48 and 72h, respectively (Fig 5B).

At 37°C and pH 5.5, the parasites were not mobile after 24h of encapsulation. The survival percentage of parasites trapped inside liposomes containing RPMI-dextran was 86.7% after 24h and 65.4% after 48h (Fig 5C). The average number of living parasites per liposome was 1.3, both after 24 h and 48h (Fig 5D). After 72h, it was not possible to quantify the viability of parasites, because most liposomes had lost a large part of their internal volume and had shrunk. The loss of internal volume indicates that water molecules, which are membrane-permeant, diffused out of liposomes. The driving mechanism for this diffusion is the necessity to balance the osmotic pressure between the RPMI-dextran solution inside liposomes and the external glucose solution. The osmotic pressure linearly varies with the total molecular concentration of solutes in the solution. Therefore, a loss of solute molecules in RPMI-dextran within liposomes induces a diffusion of water out of liposomes in order to keep constant the RPMI-dextran molecular concentration. It therefore suggests that at pH 5.5 at 37°C the encapsulated parasites consume molecules present inside the liposomes. This phenomenon is not seen at 24°C and might indicate the start of promastigote transformation towards the amastigote form.

Parasite lifetime within giant liposomes is therefore well adapted to the study of the intracellular life cycle of *L*. *amazonensis* since it is comparable to the time required *in vivo* for promastigote-to-amastigote transformation [8].

## Discussion

We have successfully produced giant liposomes that encapsulated living parasites. Several questions arise concerning the composition of these liposomes in order to be physiologically more relevant. First, for the sake of simplicity we have used commercial lipids, but the production of parasite-encapsulating liposomes prepared from lipids extracted from macrophage membrane should not raise problems. Indeed, we already produced giant liposomes (without parasites in the first step) made of these lipids (data not shown). It however requires a significant effort to extract large amount of lipids from macrophages. Moreover, this new method enabled us to produce liposomes with charged lipids that are essential components of the plasma membrane. Proteins can also been added to the liposome membranes as shown by Yanagisawa et al. [18], where active potassium channel KcsA was reconstituted in similar liposomes.

Concern around the possible trapping of oil between the two lipid leaflets of the membrane has consistently arisen for the preparation of liposomes by the inverted emulsion method. Indeed, when giant liposomes are prepared using the cDICE method [17], with a centrifugation step [16], or with jetting [20], small amounts of oil can be trapped in the membrane, and this remaining oil accumulates into discrete droplets in the membrane easily observed under phase contrast microscopy. In the present study, small droplets have never been observed in the membrane of parasite-encapsulating liposomes formed using the described variant of the ‘inverted emulsion’ method.

A quantitative study of the biophysical and biochemical parameters that control the fate of parasites encapsulated in artificial liposomes was beyond the scope of this paper. It would require investigating the role played by numerous parameters such as temperature, pH, nutrient delivery, metabolic waste removal and their coupled dynamics, on amastigote transformation and parasite replication. The experiments presented in this study clearly show that the amount of RPMI encapsulated in an artificial liposome is sufficient to ensure the survival of parasites for 96 hours and that the metabolic waste generated by a parasite during this time does not kill it. Temporal evolution of the surface area and of the tension of the liposome membrane with parasite maturation is accessible by micropipette experiments. Moreover, this approach opens the way to sequentially supply nutrients, ions or membrane proteins into the artificial PV by inducing its fusion with vesicles loaded with suitable molecules. These fusion events might be stimulated using negatively charged artificial PVs and positively charged nano-vesicles. In this case, the presence of residual oil in the liposome membrane might be an advantage since the oil could fill the hydrophobic voids created by created by the lipid reorganization during membrane fusion [21]. For example, Richmond et al. (20) who enclosed some residual oil in liposome membranes during their preparation, managed to insert functional proteins into the membrane, among which SNARE proteins. Here, SNARE proteins are of particular interest for *Leishmania* parasites since they are involved in intracellular membrane trafficking and may play an active role in the regulation of fusion events between the PV and the intracellular vesicles of the host cell.

## Conclusion

We propose a new method to trap living *Leishmania* parasites in artificial lipid vacuoles of tunable size, composition and biophysical properties. The viability of the encapsulated parasites is long enough to enable the study of intravacuolar amastigogenesis and replication. In particular, our system opens the way to a controlled nutrient delivery to the parasites by fusion events induced between parasite-encapsulating liposomes and artificial vesicles loaded with specific molecular cargoes. We believe that this approach can provide a valuable tool to investigate the minimal elements required for amastigogenesis and replication processes of *Leishmania* intracellular life-stage and to highlight the active role of the parasite in PV maturation.

## Supporting Information

S1 Movie Promastigote *L*. *amazonensis* encapsulated in liposomes right after encapsulation, at pH 7.5 at 24°C. (MP4)

S2 Movie *L*. *amazonensis* can survive 48 hours inside liposomes. Liposome containing 2 parasites in RPMI-dextran after 48 h at pH 5.5 at 24°C.
(MP4)

S3 Movie *L*. *amazonensis* can survive 96 hours inside liposomes. Liposome containing 2 parasites in RPMI-dextran after 96 h at pH 6.5 at 24°C.
(MP4)

## References

[1] Dermine JF, Scianimanico S, Prive C, Descoteaux A, Desjardins M. Leishmania promastigotes require lipophosphoglycan to actively modulate the fusion properties of phagosomes at an early step of phagocytosis. Cell Microbiol. 2000;2(2):115–26 11207568 10.1046/j.1462-5822.2000.00037.x

[2] Descoteaux MDaA. Phagocytosis: Microbial Invasion: Microbial Invasion In: Gordon S, editor. Phagocytosis: Microbial Invasion: Microbial Invasion. Advances in Cell and Molecular Biology of Membranes and Organelles. 6: Elsevier; 1999 p. 380.

[3] Kierszenbaum F, Knecht E, Budzko DB, Pizzimenti MC. Phagocytosis: a defense mechanism against infection with Trypanosoma cruzi. J Immunol. 1974;112(5):1839–44. 4361979

[4] Nichols BA, O'Connor GR. Penetration of mouse peritoneal macrophages by the protozoon Toxoplasma gondii. New evidence for active invasion and phagocytosis. Lab Invest. 1981;44(4):324–35. 7206629

[5] Descoteaux A, Turco SJ. Glycoconjugates in Leishmania infectivity. Biochim Biophys Acta 1999;1455:341–52 10571023 10.1016/s0925-4439(99)00065-4

[6] Besteiro S, Williams RA, Coombs GH, Mottram JC. Protein turnover and differentiation in Leishmania. International journal for parasitology 2007;37:1063–75 17493624 10.1016/j.ijpara.2007.03.008 PMC2244715

[7] Landfear SM. Nutrient transport and pathogenesis in selected parasitic protozoa. Eukaryotic cell 2011;10:483–93 10.1128/EC.00287-10 21216940 PMC3127635

[8] Antoine JC, Prina E, Lang T, Courret N. The biogenesis and properties of the parasitophorous vacuoles that harbour Leishmania in murine macrophages. Trends Microbiol. 1998;6(10):392–401. 9807783 10.1016/s0966-842x(98)01324-9

[9] Burchmore RJ, Barrett MP. Life in vacuoles—nutrient acquisition by Leishmania amastigotes. Int J Parasitol. 2001;31(12):1311–20. 11566299 10.1016/s0020-7519(01)00259-4

[10] Chakraborty P, Sturgill-Koszycki S, Russell DG. Isolation and characterization of pathogen-containing phagosomes. Methods Cell Biol. 1994;45:261–76. 7707990 10.1016/s0091-679x(08)61856-7

[11] Shevchuk O, Steinert M. Isolation of pathogen-containing vacuoles. Methods Mol Biol. 2013;983:419–29. 10.1007/978-1-62703-302-2_23 23494321

[12] Vinet AF, Descoteaux A. Large scale phagosome preparation. Methods Mol Biol. 2209;531:329–46. 10.1007/978-1-59745-396-7_20 19347326

[13] Roux A, Cappello G, Cartaud J, Prost J, Goud B, Bassereau P. A minimal system allowing tabulation with molecular motors pulling on giant liposomes. Proc Natl Acad Sci U S A. 2002;99(8):5394–9. 11959994 10.1073/pnas.082107299 PMC122780

[14] Angelova MI, Soléau S, Méléard P, Faucon JF, Bothorel P. Preparation of giant vesicles by external a.c. electric fields. Kinetics and applications. Prog. Colloid Polym. Sci. 1992; 89:127–131

[15] Abkarian M, Lartigue C, Viallat A. Motion of phospholipidic vesicles along an inclined plane: sliding and rolling. Phys Rev E Stat Nonlin Soft Matter Phys. 2001;63(4 Pt 1):041906 11308876 10.1103/PhysRevE.63.041906

[16] Pautot S, Frisken BJ, Weitz DA. Production of Unilamellar Vesicles Using an Inverted Emulsion. Langmuir. 2003;19(7):10.

[17] Abkarian M, Loiseau E, Massiera G. Continuous Droplet Interface Crossing Encapsulation (cDICE) for High Throughput Monodisperse Vesicle Design. Soft Matter. 2011;7:15

[18] Yanagisawa M, Iwamoto M, Kato A, Yoshikawa K, Oiki S. Oriented reconstitution of a membrane protein in a giant unilamellar vesicle: experimental verification with the potassium channel KcsA. J Am Chem Soc. 2011;133(30):11774–9 10.1021/ja2040859 21702488

[19] Tsigankov P, Gherardini PF, Helmer-Citterich M, Spa GF, Myler PJ, Zilberstein D. Regulation Dynamics of Leishmania Differentiation: Deconvoluting Signals and Identifying Phosphorylation Trends. 2014; 13 (7):1787–1799 10.1074/mcp.M114.037705 PMC4083115 24741111

[20] Richmond DL, Schmid EM, Martens S, Stachowiak JC, Liska N, Fletcher DA. Forming giant vesicles with controlled membrane composition, asymmetry, and contents. PNAS 2011; 108:9431–9436 10.1073/pnas.1016410108 21593410 PMC3111313

[21] Pincet F, Tareste D, Amar MB, Perez E. Spontaneous and reversible switch from amphiphilic to oil-like structures. Phys Rev Lett. 2005;95(21):218101 16384186 10.1103/PhysRevLett.95.218101

